# The role of transforming growth factor beta in bicuspid aortic valve aortopathy

**DOI:** 10.1007/s12055-023-01513-8

**Published:** 2023-05-25

**Authors:** Nimrat Grewal, Onur Dolmaci, Arthur Klautz, Juno Legue, Antoine Driessen, Robert Klautz, Robert Poelmann

**Affiliations:** 1 Department of Cardiothoracic Surgery, Amsterdam University Medical Center, Amsterdam, the Netherlands; 2 Department of Cardiothoracic Surgery, Leiden University Medical Center, Leiden, the Netherlands; 3 Department of Anatomy and Embryology, Leiden University Medical Center, Leiden, the Netherlands; 4 Institute of Biology, Animal Sciences and Health, Leiden University, Leiden, the Netherlands; 5 Department of Cardiology, Leiden University Medical Center, Leiden, the Netherlands

**Keywords:** Bicuspid aortic valve, TGF-β, Thoracic aortopathy, Molecular biology, Embryology

## Abstract

A bicuspid aortic valve (BAV) is the most prevalent congenital cardiac deformity, which is associated with an increased risk to develop a thoracic aortic aneurysm and/or an aortic dissection as compared to persons with a tricuspid aortic valve. Due to the high prevalence of a BAV in the general population and the associated life-long increased risk for adverse vascular events, BAV disease places a considerable burden on the public health. The aim of the present review is to discuss the role of transforming growth factor beta (TGF-β) signaling in the development of the vascular wall and on how this complex signaling pathway may be involved in thoracic aortic aneurysm formation in tricuspid and BAV patients.

## Introduction

A bicuspid aortic valve (BAV) is the most prevalent congenital cardiac deformity defined by an aortic valve which consists of two, instead of the normal three, leaflets. BAV patients have an 80-fold increased risk to develop a thoracic aortic aneurysm and/or an aortic dissection as compared to persons with a tricuspid aortic valve (TAV) [1–4]. An aortic aneurysm is a pathological widening of the aorta, which according to its location can be classified as thoracic or abdominal. Due to the high prevalence of a BAV in the general population and the associated life-long increased risk for adverse vascular events, BAV disease places a considerable burden on the public health.

To prevent the occurrence of lethal acute aortic complications, current aortic surgical guidelines recommend prophylactic vascular replacement surgery in BAV patients based on the size and progression rate of the aortic diameter [5, 6]. Aortic dissections however frequently occur in aortic dimensions below the recommended surgical threshold [7, 8]. The geometrical criterion to intervene thus is not sufficient to timely identify patients with an increased risk for thoracic aortopathy [7, 9–11]. Considering the limitations of diameter-based risk assessment in BAV, there is an unmet medical need to identify genetic and/or molecular markers to improve patient-tailored treatment strategies, including pharmaceutical stabilization. So far, no drugs have been found that can halt aneurysm growth to prevent aortic events such as dissection or rupture. Consequently, identification of the pathogenetic mechanisms leading to thoracic aortopathy is essential.

A BAV is a complex congenital malformation, with genetically an autosomal dominant inheritance pattern with reduced penetrance and variable expressivity. BAV is therefore characterized by a heterogeneous phenotype. Many studies have focused on the clinical sequelae in BAV related to the genetic background [12–15], hemodynamics [16–19], animal models [20, 21], and biomarkers [22–25]. Despite enormous efforts and great progress in research in the abovementioned areas, the exact underlying pathological pathways leading to aortic dilatation remain unknown. Connective tissue disorders, such as Marfan syndrome and Ehlers-Danlos, are monogenic syndromes which are also associated with an increased risk for aortopathy. These conditions are characterized by an increased transforming growth factor beta (TGF-β) activity [26]. Some similarities in ascending aortic wall pathology have recently been noted between BAV patients and patients with Marfan syndrome [27–29]. Considering these overlapping pathological mechanisms, it is interesting to comprehend the role of TGF-β in BAV aortopathy, which has not yet been elucidated. Stressing the need to understand the substrate of aortic complications in BAV patients, this review focuses on the fundamental ascending aortic wall pathology in BAV aortopathy and our current understanding of the role of TGF-β. We present a detailed histopathological overview of the ascending aortic wall in healthy, non-dilated and in pathologically dilated TAV individuals and compare those to the pathological conditions as seen in the BAV patients. We discuss the role of TGF-β in the development of thoracic aortopathy.

## The ascending aortic wall development and histology in the tricuspid aortic valve

### The intimal layer in tricuspid aortic valve

**Fig. 1:**
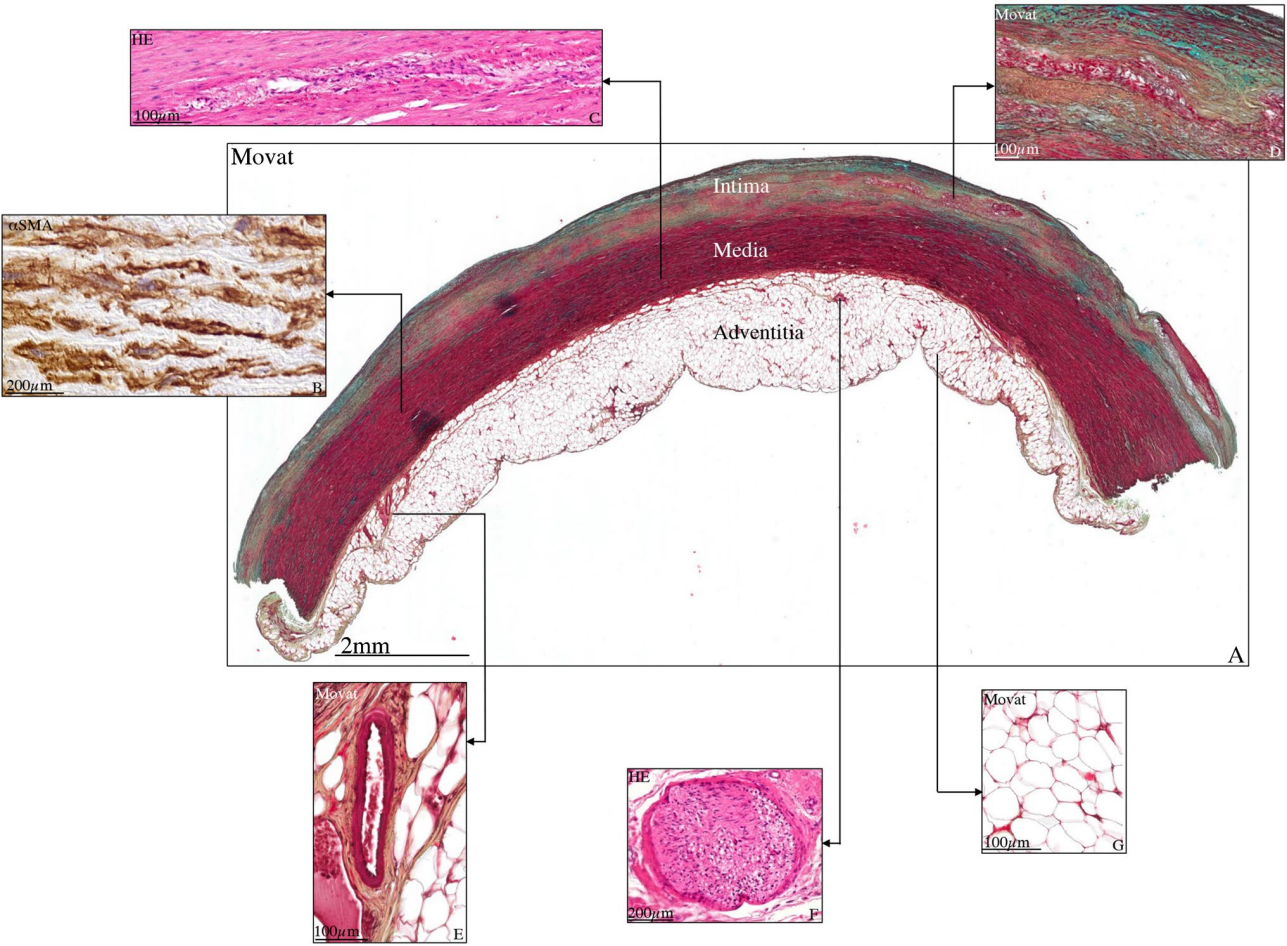
Transverse histological section of a non-dilated tricuspid ascending aorta. Sections (5 μm) are stained with Movat pentachrome staining (**A**, **D**, **E**, **G**), alpha smooth muscle actin (**B**), and hematoxylin eosin (**C**, **F**). The intima, media, and adventitia are depicted in the overview figure. Details of the vascular components are shown in the inserts. The vascular SMCs are shown in the alpha smooth muscle actin–stained section (**B**), vasa vasorum in the medial layer is shown in the hematoxylin and eosin–stained section (**C**), and an atherosclerotic plaque in the intimal layer is highlighted in the Movat-stained section in **D**. **E** A detailed view of vasa vasorum in the adventitial layer. A nerve cell (**F**) and clustered adipocytes (**G**) are further shown in a hematoxylin and eosin- and Movat-stained sections, respectively. HE, hematoxylin and eosin; αSMA, alpha smooth muscle actin; SMC, smooth muscle cell. Scale bars shown in figure

**Fig. 2:**
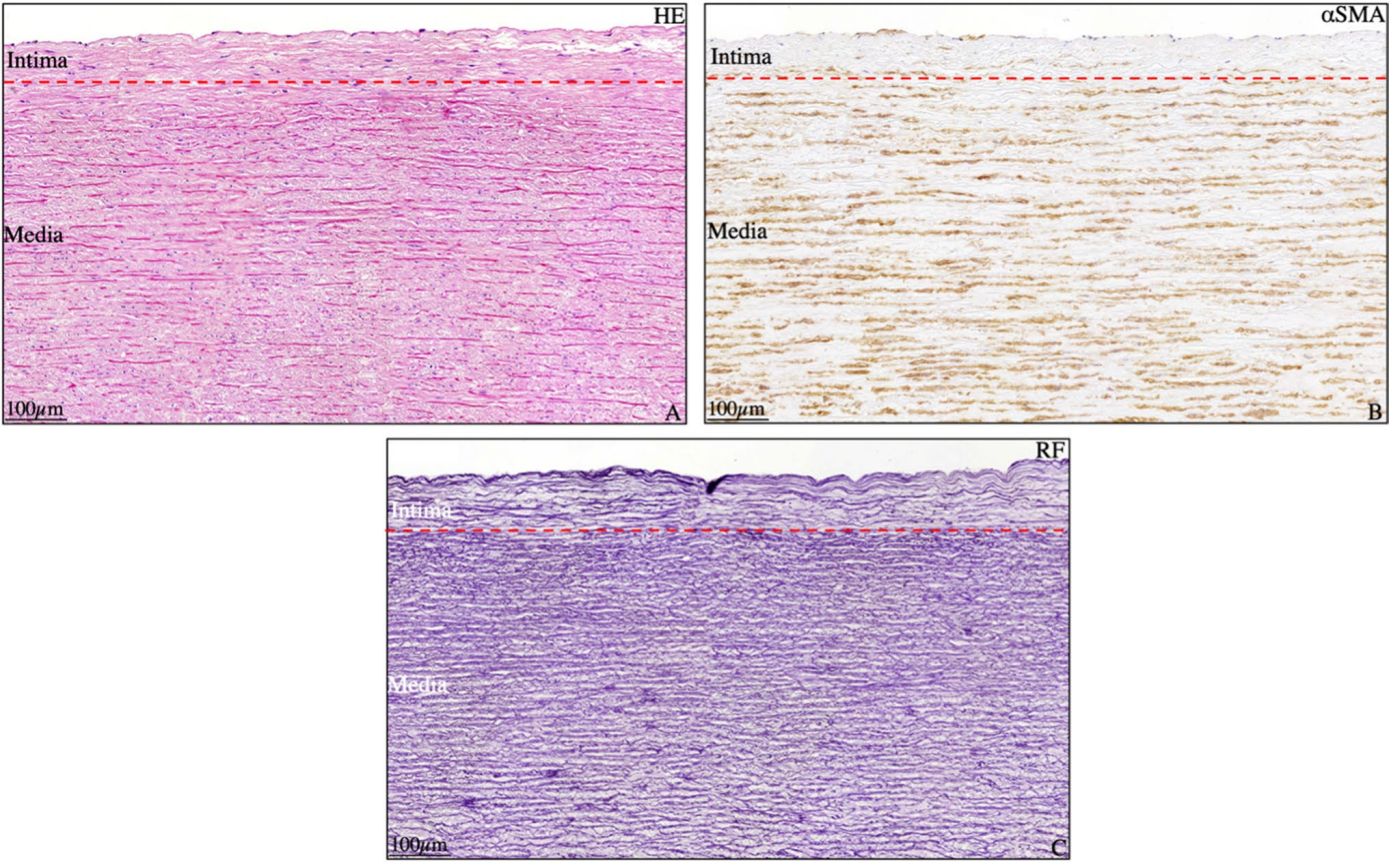
Transverse histological Section (5 μm) of a tricuspid non-dilated ascending aortic wall stained with hematoxylin and eosin (**A**), alpha smooth muscle actin (**B**), and resorcin fuchsin (**C**). The intimal layer is depicted with the red dashed line. The intima is filled with fine elastic lamellae and is devoid of alpha smooth muscle actin expression. HE, hematoxylin and eosin; αSMA, alpha smooth muscle actin; RF, resorcin fuchsin. Scale bars shown in figure

The aorta is an elastic artery consisting of three layers: the tunica intima, media, and adventitia (Fig. 1). The intima is the innermost layer and endothelial cells form the border of the luminal surface. The formation of the intima starts in the early phases of embryonic development. It has been demonstrated that the intimal layer in a TAV individual at 10 weeks gestational age consists of a single endothelial cell layer which is closely adherent to a thick internal elastic lamina (lamina elastica interna). The internal elastic lamina separates the intimal layer from the middle layer of the ascending aorta [30]. The intimal layer contains only a single elastic lamina until the age of 4 months, after which the intimal thickness increases between the endothelial layer and the internal elastic lamina, forming the so-called sub-endothelial layer. This sub-endothelium consists of fine, thin, and fragmented elastic fibers, with large amounts of extracellular matrix, particularly collagen types I and III and is devoid of vascular smooth muscle cells (SMCs) until the age of 6 months [31]. During growth, a further increase in thickness of the intimal layer is seen [30], due to a process of low-grade injury and repair (Fig. 2) [32]. It is believed that vascular injuries transform vascular SMCs from a metabolic quiescent but contractile to a synthetic and proliferative phenotype through a process called phenotypical switching [33]. This differentiation of vascular SMCs forms an integral part in embryonic vascular development and adult vascular pathology. TGF-β signaling plays pivotal roles in SMC differentiation during vascular development as well as phenotypic switching in disease states [34]. TGF-β is the founding member of the TGF-β superfamily that comprise TGF-βs, activins, bone morphogenetic proteins (BMPs), and growth and differentiation factors (GDFs) [35]. The TGF-β signaling pathway is regulated in multiple steps by several factors which can be summarized as the canonical and non-canonical pathway [36]. In the canonical pathway, TGF-β function is directly mediated by the transcription of Suppressor of Mothers Against Decapentaplegic (SMAD)–dependent target genes. In the non-canonical pathway, TGF-β function is mediated by SMAD-independent pathways including mitogen-activated protein kinase (MAPK) signaling pathways and Wingless-related integration (Wnt) signaling.

The thickened intimal layer consists of extracellular matrix proteins and vascular SMCs besides fine elastic lamellae. Ageing may further lead to the development of atherosclerosis in the intimal layer, a chronic progressive inflammatory disease which can be variably present and in different stages (Fig. 1) [37]. Studies using lineage tracing and vascular SMC–specific manipulation have suggested that phenotypic switching of SMCs might also play a role in atherosclerosis and plaque stability [33]. The role TGF-β plays in the development of atherosclerosis has not yet been fully elucidated. According to recent literature, this signaling pathway cannot be considered as fully “atheroprotective” or “atherogenic”; TGF-β is more likely to play a central role in both normal and pathological vascular repair [38].

### The medial layer in tricuspid aortic valve

The medial layer is defined as the area between the internal elastic lamina and the outermost layer, the adventitia. In the premature TAV, the medial layer consists of elastic lamellae without any form of pathology such as elastic fiber thinning, fragmentation, or degeneration. The number of lamellae in the media increases significantly between the premature and neonate life phase. Interestingly, the number of lamellae increases until individuals are 6 years old, after which the number stabilizes for a few years [30, 32].

In adulthood, the number decreases significantly [30, 39].

**Fig. 3:**
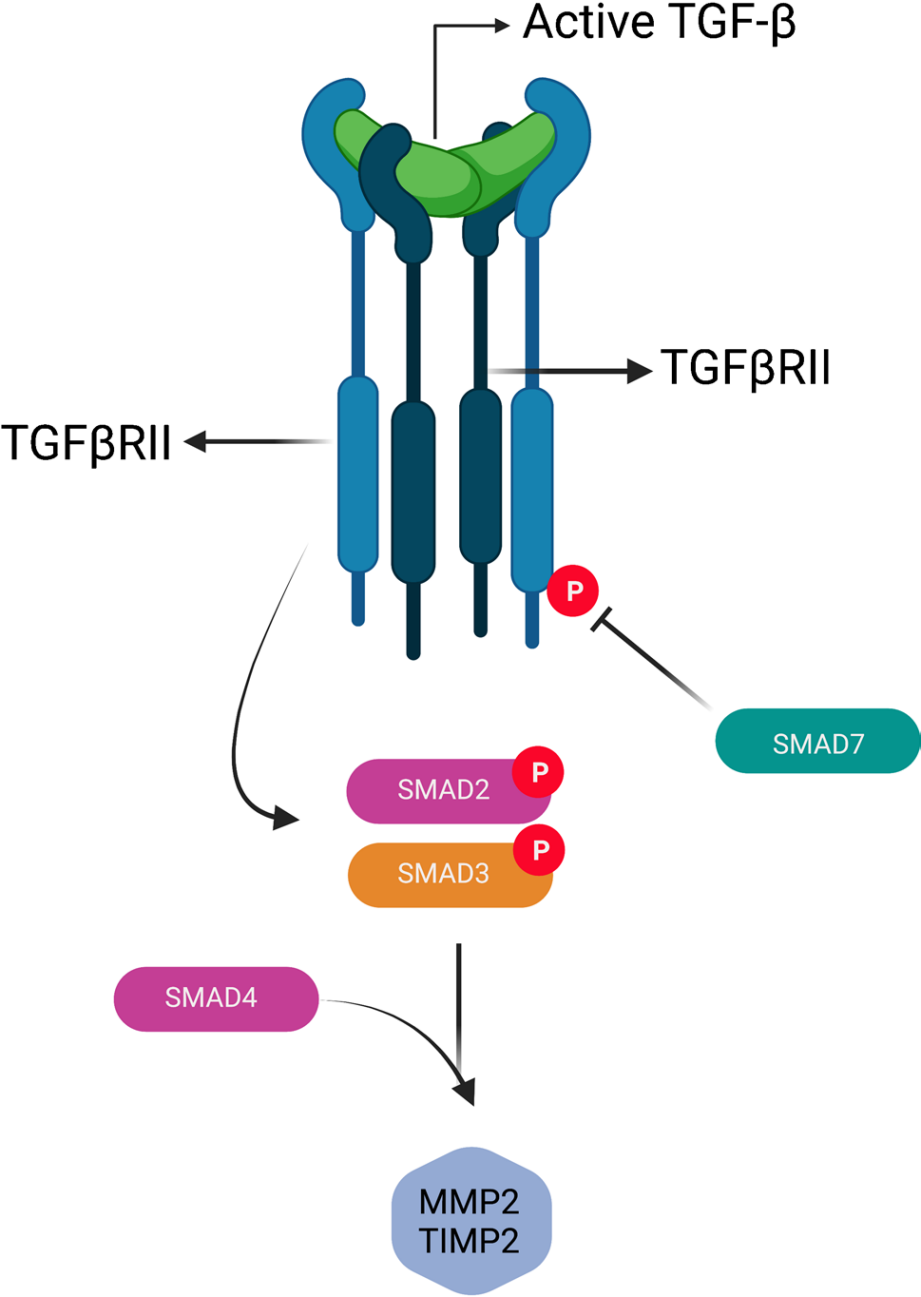
Schematic overview of the transforming growth factor beta (TGF-β) signaling pathway linked to matrix metalloproteinase (MMP) production. Activated TGF-β from the latent complex binds to its cell surface receptor II (TGFβRII), inducing the activation of TGF-β type I receptor (TGFβRI). The then activated TGFβRI phosphorylates SMAD2,3. SMAD7 on the other hand inhibits the TGF-β signaling at the receptor level. Phosphorylated SMADs interact with co-SMAD4 which induces the production of MMP2 and tissue inhibitors of matrix metalloproteinases (TIMPs). Created with Biorender.com

Apart from elastic lamellae, the medial layer consists of extracellular matrix, vascular SMCs, and vasa vasorum [30, 40]. With increasing age, a decrease of the elastin to collagen ratio is seen in the aorta. The elastin content decreases, whereas other matrix components, primarily collagen which is absent to rare in young aortas, increase [41–43]. This process of vascular remodeling alters the vessel wall architecture in response to hemodynamic changes or various forms of vascular injury and serves to maintain the aortic diameter and consistent blood flow under normal physiological conditions. Matrix metalloproteinases (MMPs) play an important role in vascular remodeling and control degradation of extracellular matrix proteins, such as elastin and collagen. Histopathological analysis of tricuspid aneurysmal tissue has shown a characteristic pathological remodeling of collagen and elastin, driven by enhanced production of extracellular proteases and loss of vascular SMCs. The integrity of the aortic extracellular matrix may therefore be compromised by an enhanced MMP activity. Overactivity of MMPs is possible by two means: either due to overproduction by vascular smooth muscle and inflammatory cells in response to vascular injury inflammation, oxidative stresses, matrix degradation products or due to an increased TGF-β activity [44, 45] (Fig. 3).

The major constituents of the aortic media are the vascular SMCs. These are highly specialized cells with contraction and regulation of blood vessel tone-diameter as their main function. During development, SMCs play a key role in the morphogenesis of blood vessels and exhibit high rates of proliferation and production of extracellular matrix components (i.e., collagen, elastin, and proteoglycans), while at the same time acquiring contractile properties [33]. Several studies have demonstrated the importance of TGF-β signaling pathway in SMC differentiation during embryonic development [35]. Adult vascular SMCs are not terminally differentiated and retain the possibility to undergo a phenotypic switch in response to local environmental cues [46]. In adult human blood vessels, the vascular SMCs are characterized by a low rate of proliferation/turnover and a very low rate of synthesis of extracellular matrix components and thus are completely dedicated to carry out their contractile function. However, in response to vascular injury, SMCs dramatically increase their rate of proliferation, migration, and synthetic capacity, playing a crucial role in vascular repair [33, 47]. The ability to phenotypically switch between a “contractile” and “synthetic” phenotype is an important feature of the differentiated vascular SMCs. As discussed above, TGF-β plays a major role in their ability to differentiate and switch between phenotypical states. The possibility to undergo a phenotypical switch in response to environmental cues also plays a major role in the development of cardiovascular diseases, such as atherosclerosis, hypertension, and aneurysm formation [47–49]. As mentioned above, in TAV individuals, the number of vascular SMCs decreases in the ageing aorta through apoptosis in the aortic media [39, 42].

### The adventitial layer in tricuspid aortic valve

The media to adventitia border is marked by the last, outermost elastic lamella of the media, the *external* elastic lamina. The adventitia is the surrounding loose fibrous layer that contains connective tissue, the vasa vasorum, adipocytes, and nerve cells (Fig. 1). This layer varies in thickness and composition depending on the site of the ascending aorta, being thick at the aortic root level and thin in the epicardium-covered part of the ascending aorta inside the pericardial cavity. Its variable thickness across the ascending aorta and its rich collagen content give the adventitia the greatest tensile strength of the three aortic wall layers [32]. Distal to the pericardial reflection, the adventitia merges with the surrounding mesenchyme of the thorax. The vasa vasorum normally extends into the outer third of the medial layer. Extension of the vasa vasorum deeper into the media helps to preserve the integrity of the vascular SMCs with ageing, as diffusion of reagents from the lumen into the vascular SMCs becomes difficult with increasing size [50].

## The ascending aortic wall development and histology in the bicuspid aortic valve

Patients with a BAV often develop life-threatening thoracic aortopathy. Histopathological differences have mainly been concluded by studying dilated end-stage ascending aortic specimen in BAV and TAV. Recently, focus has shifted towards the structural make-up of the non-dilated ascending aortic wall in BAV patients [37]. These studies analyzed the ascending aortic wall in the premature and adult phase and revealed several differences between BAV and TAV patients [30, 37].

### The intimal layer in bicuspid aortic valve

**Fig. 4:**
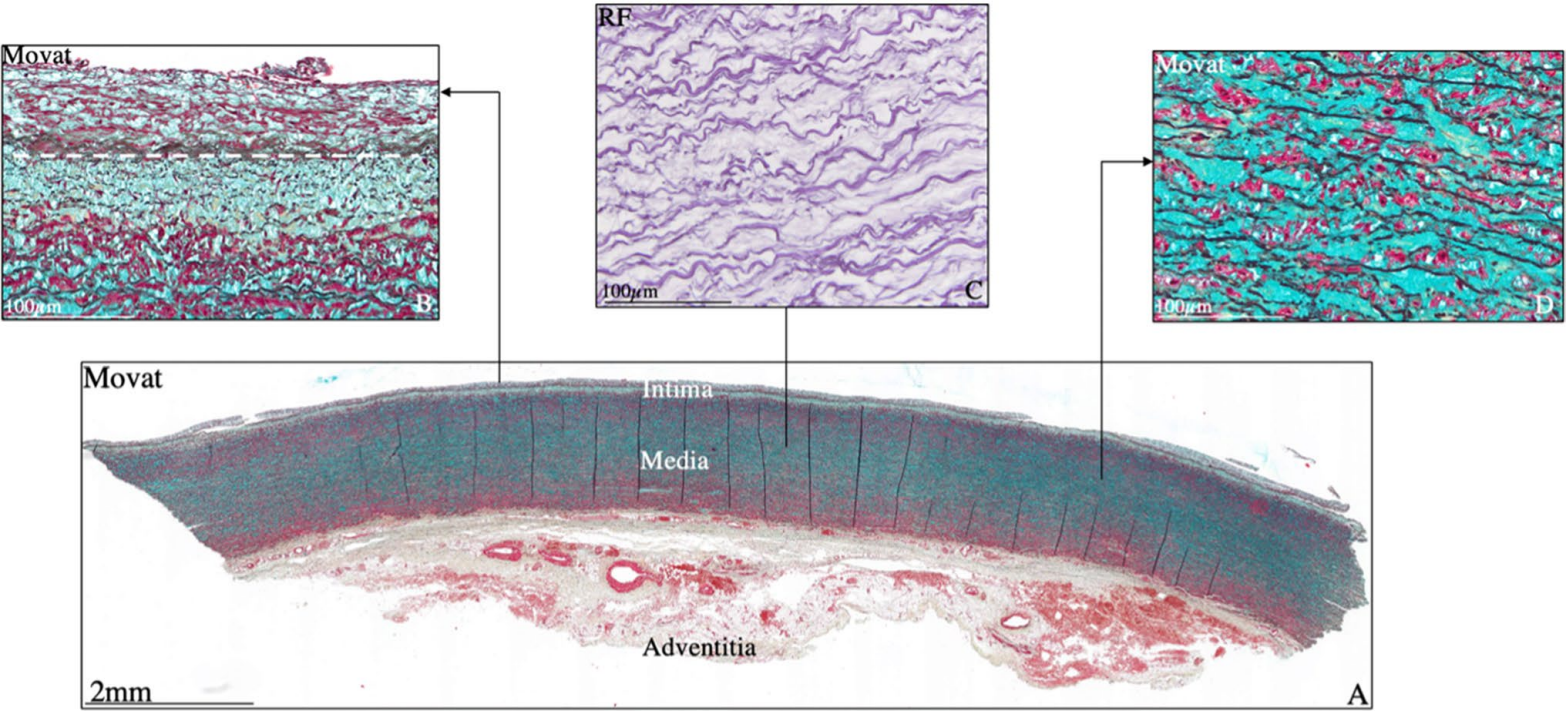
Transverse histological section (5 μm) of a dilated bicuspid ascending aorta (Movat pentachrome staining — **A**, **B**, **D** and resorcin fuchsin staining — **C**). The intima, media, and adventitia are depicted in the overview figure. Details of the vascular components are shown in the inserts. The intimal layer is shown in Fig. 4B; the white dashed line depicts the border between the intima and the media. The fine elastic lamellae in the media are shown in Fig. 4C, with hardly any fragmentation. In Fig. 4D, the Movat-stained medial layer shows elastic lamellae in black, vascular smooth muscle cells in red, and an abundance of mucoid extracellular matrix in blue between the elastic lamellae. RF, resorcin fuchsin. Scale bars shown in figure

The intimal layer in the premature BAV comprises an endothelial cell layer and internal elastic lamina. In contrast to the TAV however, the sub-endothelial layer already shows thickening before birth [30], leading to a significantly thicker intimal layer. Comparable to the TAV, the sub-endothelial layer in the premature BAV consists of fine elastic lamellae and some extracellular matrix. No intimal vascular SMC expression is seen at this age. The intimal layer becomes significantly thinner directly after birth, with only a single internal elastic lamina in both the non-dilated and dilated BAV patients (Fig. 4A, B). Interestingly, a significantly thin intimal layer has also been described in patients with Marfan syndrome [28, 29]. Marfan syndrome is characterized by a defect in the TGF-β signaling pathway. Considering the prominent role of TGF-β in the development of the intimal layer, it is plausible that in the BAV patients, a defect in the TGF-β signaling pathway is responsible for the lack of intimal thickening after birth and during their lifetime. Our previous work has further shown that the intimal layer in the BAV is completely devoid of TGF-β expression and the downstream signaling factor phosphorylated SMAD2 (pSMAD2), in both the non-dilated and dilated ascending aortic wall, whereas the expression was significantly higher in all TAV patients. Clinical and histopathological studies have also shown that the BAV ascending aorta does also not show any signs of (age-dependent) atherosclerosis, which is commonly seen in the TAV [13, 14, 37].

### The medial layer in bicuspid aortic valve

The premature BAV medial layer consists of elastic lamellae, vascular SMCs, and mucoid extracellular matrix, which is similar to the TAV. Differences are however noted in the development of the vascular wall elements. Elastic lamellae in BAV are significantly thinner in both the non- and dilated BAV and hardly show any fragmentation (Fig. 4C). The number of lamellae remains stable throughout most of their lifetime, without observing a decrease in adulthood or difference between non-aneurysmal and aneurysmal tissue.

Vascular SMCs are critical in the differences between BAV and TAV histopathology. Not only have these cells been found in apoptosis [51, 52], they are also morphologically distinct in the BAV population. Vascular SMCs in BAV patients are less well differentiated in both non-dilated and dilated BAV patients as revealed by a significantly lower expression of differentiated vascular SMC markers: smoothelin, calponin, and SM22alpa. Furthermore, Lamin A/C, playing a key role in the differentiation of vascular SMCs, is significantly expressed lower in the non-dilated and dilated BAV as compared to the TAV population [53, 54]. Their immaturity is noted in both the non- and dilated BAV population, excluding aortic dilatation as the consequence of the observed phenotypic switch of the vascular SMCs. Recent studies have emphasized that an early developmental defect could be held responsible for the formation of both a BAV and related aortic wall complications [55].

During embryogenesis, the heart and vessels are the first organ to form. The cardiac development is characterized by a staged formation of all elements which contribute to the normal functioning of the heart. The valves are formed between the fifth and the eight weeks of fetal life. Three distinct cardiac progenitor cells are involved in the formation of the semilunar valves through a process of endocardial cushion formation, being the neural crest, second heart field, and endocardial cushion–derived cells [55, 56]. The three leaflets of the aortic valve are expected to have a different origin: the two main facing cushions are filled by mesenchymal cells, derived both from the endocardium, and later arriving neural crest cells. The third leaflet has a more second heart field–related etiology [57]. The vascular SMCs of the great arteries are also derived from the abovementioned progenitor cell lines. An early embryonic defect could therefore simultaneously lead to a malformed aortic valve and immaturity of the vascular SMCs. Murine genetics studies have shown that a loss of TGF-β signaling components generally leads to abnormal differentiation and maturation of the primitive vascular network, including a failure of SMC recruitment and/or differentiation [58]. Medial accumulation of mucoid extracellular matrix components is significantly higher in non-dilated and dilated BAV patients as compared to the TAV (Fig. 4D) [37, 59].

### The adventitial layer in bicuspid aortic valve

The BAV adventitial layer consists of similar elements as seen in the TAV population, being loose fibrous tissue, large nerve fibers, vasa vasorum, and adipocytes. Differences in the vasa vasorum function in BAV have been suggested recently. The adventitia varies in thickness in the BAV too, independent of age and gender.

Histopathological findings in tricuspid, bicuspid and Marfan syndrome patients are summarized in Table 1.

**Table 1:** Histopathological findings in tricuspid, bicuspid and Marfan syndrome patients

	Tricuspid aortic valve	Bicuspid aortic valve	Marfan syndrome
Tunica intima	• Thick intimal layer • Atherosclerosis + • Histopathologically TGF-β and pSMAD2 expression +	• Significantly thinner intimal layer • Atherosclerosis – • Defective TGF-β signaling • Histopathologically TGF-β and pSMAD2 expression –	• Significantly thinner intimal layer • Atherosclerosis – • Defective TGF-β signaling • Histopathologically • TGF-β and pSMAD2 expression –
Tunica media	• Differentiated VSMCs • TGF-β expression +	• Immature VSMCs • Loss of TGF-β expression	• Immature VSMCs • Loss of TGF-β expression
Tunica adventitia		• Reported difference in vasa vasorum	

Legend: *pSMAD2*, phosphorylated SMAD2; *TGF-β*, transforming growth factor beta; *VSMCs*, vascular smooth muscle cells

## Discussion

A BAV is the most common congenital cardiac malformation, which carries an extremely high risk for thoracic aortic aneurysm development. Other conditions which also carry an increased susceptibility for thoracic aortopathy are related to specific genetic conditions, such as Marfan syndrome, Loeys-Dietz syndrome, Ehlers-Danlos syndrome, and familial thoracic aortic aneurysms and dissections. Thoracic aortopathy is however most commonly seen in patients with a TAV, in the form of degenerative thoracic aortopathy. Many studies have recently compared the ascending aortic wall pathology in abovementioned conditions and discovered specific histopathological features in the aortic wall make-up. The disease-of-interest, thoracic aortopathy, is a very heterogeneous disorder with multiple distinct underlying genetic mutations but common clinical phenotypes and histopathologic and molecular findings [27, 53, 60, 61]. As a thoracic aortic aneurysm does not have any preceding symptoms, the first manifestation often is chest pain due to a ruptured aorta. Management of thoracic aortopathy therefore remains a challenge in elective as well as emergency cases. The identification of common pathological mechanisms would significantly improve the individual cardiovascular risk stratification and thus will result in a major improvement in the field of personalized medicine. The aim of the present review is to discuss the role of TGF-β signaling in both the development of the vascular wall and on how this complex signaling pathway may be involved in thoracic aortic aneurysm formation in TAV and BAV patients.

The TGF-β signaling pathway not only plays a crucial role in vascular development, but also in degenerative thoracic aortic aneurysm formation in the TAV population. Thoracic aortopathy develops as a result of maladaptive remodeling of the vascular extracellular matrix. In normal conditions, a balance exists in vascular remodeling with matrix deposition and degradation resulting in maintenance of the structural integrity of the vascular wall. Recent evidence highlights that a dysregulation of TGF-β signaling disrupts the balance in favor of enhanced proteolysis resulting in pathological remodeling of the vascular extracellular matrix [62]. A dysregulation of the TGF-β canonical signaling pathway has further been shown to lead to a fragmentation of the elastic lamellae [63] causing weakening of the aortic architecture and increased susceptibility for aortic dilatation and dissection [64]. In the bicuspid research field, recent focus has shifted towards a common embryonic origin of both valvular and vascular development. Genetic defects in early development can account for both a deformed aortic valve and defective composition of the ascending aortic wall. Progenitor cells are responsible for region-specific vascular SMCs in the aortic wall. Therefore, defects in the neural crest and second heart field signaling in the BAV patients lead to BAV-related aortopathy in the proximal thoracic aorta that is rarely found in the descending thoracic aorta [27]. In comparison in Marfan patients, defects are seen in the neural crest, second heart field, and paraxial mesoderm which cause aortopathy in both the ascending and descending aorta. In this review, we discussed the characteristic features of the bicuspid aortic wall, including a thin intimal layer, a phenotypical switch defect resulting in less differentiated SMCs, excessive mucoid extracellular matrix accumulation, and lack of atherosclerosis. Previous studies have shown that the ascending aortic wall in Marfan syndrome and a dissected aortic wall share many features in common with the BAV, in form of a thin intimal layer, immature vascular SMCs, and lack of atherosclerosis [4, 65] (Table 1). Considering the functions of TGF-β in the development of the intimal layer and normal vascular remodeling and phenotypical switch of vascular SMCs, it is plausible that a defect of this signaling pathway is responsible for the aortic pathology encountered in patients with a BAV. Expression of TGF-β and downstream signalers has been studied in several ways. Our group investigated the expression of TGF-β and pSMAD2 in the ascending aortic wall in the non- and dilated BAV and TAV patients. We found that the intima lacked TGF-β and pSMAD expression and the medial expression was significantly lower in the BAV as compared to the TAV dilated specimen. Our findings are in line with many other studies which found a decreased expression of TGF-β in the BAV aorta as compared to the TAV [63, 66], also investigating the canonical SMAD-mediated pathway. In murine aneurysmal studies, an increased TGF-β signal through the non-canonical pathway has been described leading to extracellular matrix degradation [67, 68]. Unfortunately, special attention to the intimal layer has not been given by any other study. Besides differences in expression between valve morphologies, we were able to identify non-dilated BAVs with increased susceptibility for future complications on basis of the medial expression of TGF-β, pSMAD2, and MMP9 [25]. Ikonomidis et al. also suggested in their study that a unique profile of plasma MMPs, tissue inhibitors of MMPs, and micro ribonucleic acids (microRNAs) could possibly predict the increased risk for thoracic aortopathy in BAV and TAV using a plasma multianalyte regression strategy [24]. Expression of TGF-β and markers of the non- and canonical pathway should therefore be further investigated as a marker to distinguish BAV patients with an increased risk for future aortic complications. Differences in the amount of TGF-β in the aortic wall have further been reported to correlate well with the levels of shear stress on the wall [69]; therefore, in future studies, the role of shear stress on the aortic wall should also be considered. It is interesting to note that a specific group of animals like reptiles normally present with bicuspid semilunar valves. Evidently, these have a different physiology, being “cold-blooded” as well as anatomy, as they present two aortas [70]. Nevertheless, here the bicuspid valves function efficiently, even until advanced ages without signs of thoracic aortopathy. It would therefore be interesting to study the TGF-β expression in reptiles where BAV prevails under normal conditions.

## Conclusion and future directions

In most of the thoracic aortic aneurysm cases, an increased TGF-β activity has been identified. Characteristics in BAV point at a decreased or defective signaling in the TGF-β signaling pathway. A defect in early embryogenesis is most likely responsible for the development of both a BAV and an abnormal ascending aortic wall, which is susceptible for future aortic complication. The characteristic BAV findings, which can be related to a defective TGF-β signaling are, however, generic to all BAV patients and cannot be applied to identify the high-risk subset. Furthermore, a BAV shares many histopathological features with Marfan syndrome characterized by a defect in the TGF-β signaling pathway. In Marfan however, a BAV is not an obligatory clinical manifestation, indicating that a defective TGF-β signaling is at least not the main factor causing BAV formation. Even though many histopathological features in BAV can be explained by a decreased TGF-β activation, future studies will have to focus on differences in expression in the non-dilated BAV groups to be able to distinguish cause and effect of expression and identify patients with an increased vulnerability for future thoracic aortopathy.

## Data Availability

Not applicable.
